# Implantable cardioverter-defibrillator prevents sudden death in patients with Chagas cardiomyopathy in the Brazilian Amazon

**DOI:** 10.1590/0037-8682-0480-2020

**Published:** 2021-03-22

**Authors:** Katia do Nascimento Couceiro, Jessica Vanina Ortiz, Mônica Regina Hosannah da Silva e Silva, Débora Raysa Teixeira de Sousa, Kenny Rodrigues de Souza, Gabriela Maciel Alencar, Laylah Kelre Costa Magalhães, Maria das Graças Vale Barbosa Guerra, João Marcos Bemfica Barbosa Ferreira, Jorge Augusto de Oliveira Guerra

**Affiliations:** 1 Universidade do Estado do Amazonas, Programa de Pós-Graduação em Medicina Tropical, Manaus, AM, Brasil.; 2 Fundação de Medicina Tropical Dr. Heitor Vieira Dourado, Manaus, AM, Brasil.; 3 Fundação de Hematologia e Hemoterapia do Amazonas, Manaus, AM, Brasil.

**Keywords:** Chagas cardiomyopathy, Implantable cardioverter-defibrillator, Amazon region

## Abstract

Chagas disease (CD), with approximately 10,000 deaths annually, has become a worldwide health problem. Approximately 35% of cases may show cardiac manifestations such as arrhythmias and/or conduction disorders, heart failure, thromboembolic accidents, and sudden death. The Amazon region has long been considered a non-endemic area for CD; however, in the last decades, with an increase in the number of acute and chronic cases, disease evolution has received greater attention. Here, we report the successful implementation of a cardioverter-defibrillator for the prevention of sudden death in a patient with autochthonous Chagas cardiomyopathy in the Brazilian Amazon.

## INTRODUCTION

Chagas disease (CD) is endemic in Latin America and has become a worldwide public health problem due to the increase in human migration. It was considered a neglected disease in 2005. One hundred and ten years after its discovery, the World Health Organization now estimates that 70 million people are at risk of acquiring the disease, 7 million people are infected, and 10,000 deaths are caused by the disease annually^1^.

This disease can manifest in two stages: an acute stage in an asymptomatic or a symptomatic form, and a chronic stage in indeterminate, cardiac, or digestive forms. Approximately 60% of infected individuals have the indeterminate form, 25% to 35% develop heart disease; of these, 10% may develop severe heart disease^2^. In endemic regions, it is an important cause of sudden death. Chagas cardiomyopathy (CCM) manifests in the form of arrhythmias and/or conduction disorders, heart failure, thromboembolic accidents, and sudden death. Its pathogenesis is multifactorial and complex. Chronic inflammation can result in fibrosis and consequent sinus node dysfunction, atrioventricular and intraventricular conduction abnormalities, and ventricular tachyarrhythmias^2^ ^,^ ^3^.

The Amazon region has long been considered a non-endemic area for CD; however, in recent decades, with an increase in the number of acute and chronic cases, this profile has changed. In the state of Amazonas, the first patient with serologically positive chronic disease was recorded in 1977^3^. Since then, surveillance programs and serological surveys have been carried out, and new cases have been detected. Previous studies suggest lower morbidity levels related to CD in the Amazon region, probably due to the presence of different *T. cruzi* strains from those found in traditionally endemic areas^3^ ^,^ ^4^.

Despite this, a few cases of CCM have been reported in the Amazon. The first reports of dilated cardiomyopathy with chagasic etiology in this region date back to 2003, with two fatal cases^5^, 3 autochthonous cases diagnosed in 2009^6^, and cases of ventricular tachycardia in 2012^7^. 

In this study, we present a case report of successful cardioverter-defibrillator implantation for the prevention of sudden death in a patient with autochthonous CCM in the Brazilian Amazon. 

## CASE REPORT

A 60-year-old man, a painter currently living in the state capital, Manaus but born in Autazes in the interior of Amazonas, where he lived until the age of 18, was diagnosed with Chagas disease in 2015 after an attempt to donate blood. He developed the cardiac form of the disease and presented with dilated cardiomyopathy with ventricular dysfunction. His left ventricular ejection fraction (LVEF) was 44% using the Simpson method. An apical aneurysm was observed, and a baseline 12-lead electrocardiogram (ECG) showed an abnormal sinus rhythm and changes in ventricular repolarization in the lower-lateral wall. On the 24-hour Holter recording, he presented 16,881 isolated, bigeminated, polymorphic ventricular arrhythmias.

**FIGURE 1:**
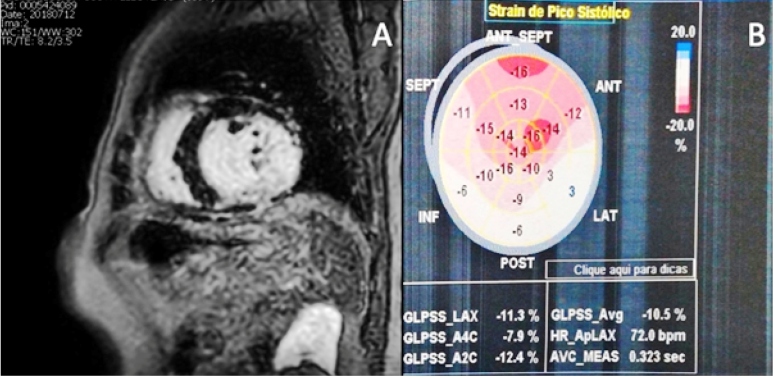
**(A)** Cardiac magnetic resonance imaging with the presence of late transmural enhancement in the lower-middle-basal wall; **(B)** Transthoracic echocardiogram with global longitudinal strain (-10.5%).

In 2018, cardiac magnetic resonance imaging was performed revealing increased cavity volumes and significant left ventricular dysfunction in addition to a late transmural enhancement in the inferior-medial-basal wall (Figure 1A). Furthermore, an echocardiogram showed a global longitudinal strain of -10.5% and a considerably low LVEF of 29% (Figure 1B). A positive high-resolution ECG for the detection of the presence of late potentials was performed, and 12% myocardial fibrosis was detected using the Selvester score^8^. 

**FIGURE 2:**
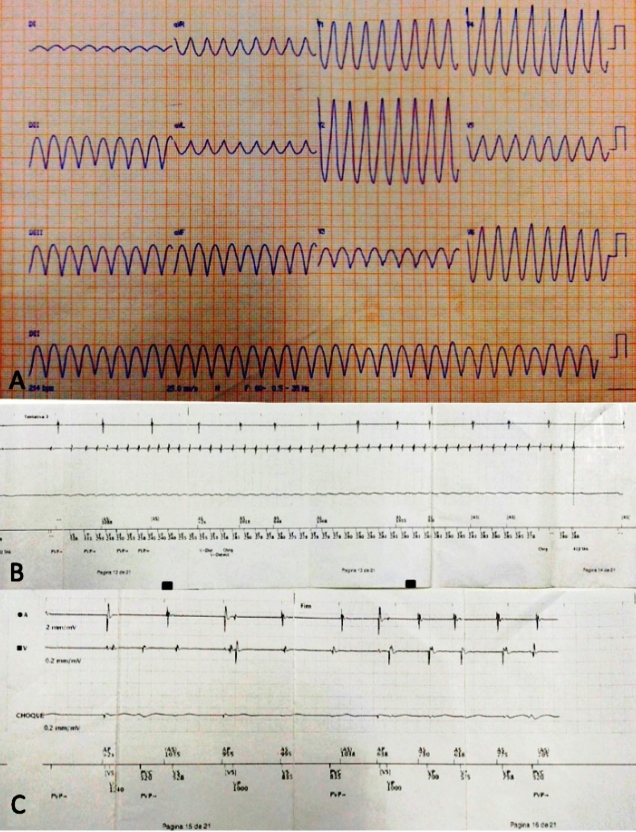
**(A)** Baseline ECG with monomorphic ventricular tachycardia without pulse; **(B)** Sustained ventricular tachycardia detected by the intracavitary electrogram; **(C)** Intracavitary electrogram stimulating the atria and ventricles after ICD shock.

In August 2019, he was admitted to the emergency department with tachycardic palpitations, cold sweats, and syncope. In the emergency room, monomorphic ventricular tachycardia without pulse was monitored and recorded on the ECG (Figure 2A), which showed cardiopulmonary arrest. He was immediately subjected to cardiac resuscitation maneuvers and was reverted to tachyarrhythmia after a 200-J biphasic shock considering he presented with isolated ventricular tachycardia.

Since the time of diagnosis of CD and CCM, the patient has been on medication optimized for heart failure: enalapril 20mg/day, carvedilol 50mg/day, and spironolactone 25mg/day. During the cardiorespiratory arrest episode with pulseless monomorphic ventricular tachycardia and after its reversal with defibrillation, the patient was referred for the implantation of an implantable cardioverter-defibrillator (ICD) as secondary prophylaxis for sudden death.

After discharge from the hospital, he was referred for outpatient follow-up and was prescribed medication for heart failure in combination with 400 mg/day amiodarone in order to avoid arrhythmic storms. Twenty-four-hour Holter monitoring was performed again, which demonstrated the absence of ventricular arrhythmias. Approximately three months after ICD implantation, the patient presented with an ICD shock episode, documented by intracavitary electrogram during telemetric evaluation (Figure 2B and 2C).

## DISCUSSION

To date, there are few published records of the occurrence of chagasic cardiomyopathy in the Amazon region, in which, ventricular aneurysms^6^ and ventricular tachyarrthythmias^7^ have been described; both of these are life-threatening clinical manifestations^9^.

Marques et al. (2012)^7^ reported the first known case of ventricular tachycardia in an autochthonous patient in the Amazon. This highlights the importance of the continuous monitoring of patients with CD in the region since the earlier the predictors of arrhythmias are identified, the lower the severity and permanent damage, the better the prognosis, the lower the cost burden on the public health system, and the better the patient's social and economic quality of life^10^.

Although both early diagnosis and monitoring are important, the patient in this report already had myocardial fibrosis identified by cardiac magnetic resonance, with late myocardial enhancement. Malignant ventricular arrhythmias are more prevalent than other forms of heart disease in patients with CD, and sudden cardiac death is commonly observed in CD; therefore, this cardiomyopathy is a frequent indication for ICD implantation^10^. 

Myocardial fibrosis identified using cardiac magnetic resonance, with late myocardial enhancement serves as an important predictor of arrhythmias and sudden death in several ischemic and non-ischemic cardiomyopathies, such as hypertrophic cardiomyopathy and CD, and it should be considered for patient monitoring and assessment of severity and interventions^11^. 

The arrhythmogenic form of CD is an important cause of mortality^2^. Although it has been infrequently reported in the Amazon region, the arrhythmogenic form of CCM is an important cause of sudden death, as evidenced in our case. 

In recent decades, the occurrences of acute and chronic cases of CD have increased significantly in regions where diagnostic and therapeutic resources are scarce. This case report reinforces the need for improvement of regional diagnostic resources, early identification of patients at greatest risk, and prevention of sudden death.
